# Inter-Cohort Validation of SuStaIn Model for Alzheimer’s Disease

**DOI:** 10.3389/fdata.2021.661110

**Published:** 2021-05-20

**Authors:** Damiano Archetti, Alexandra L. Young, Neil P. Oxtoby, Daniel Ferreira, Gustav Mårtensson, Eric Westman, Daniel C. Alexander, Giovanni B. Frisoni, Alberto Redolfi, for Alzheimer’s Disease Neuroimaging Initiative and EuroPOND Consortium

**Affiliations:** ^1^ Laboratory of Neuroinformatics, IRCCS Istituto Centro San Giovanni di Dio Fatebenefratelli, Brescia, Italy; ^2^ Department of Neuroimaging, Institute of Psychiatry, Psychology and Neuroscience, King’s College London, London, United Kingdom; ^3^ Department of Computer Science, UCL Centre for Medical Image Computing, London, United Kingdom; ^4^ Division of Clinical Geriatrics, Center for Alzheimer Research, Department of Neurobiology, Care Sciences and Society, Karolinska Institutet, Stockholm, Sweden; ^5^ Department of Radiology, Mayo Clinic, Rochester, MN, United States; ^6^ Memory Clinic and LANVIE - Laboratory of Neuroimaging of Aging, University Hospitals and University of Geneva, Geneva, Switzerland; ^7^ Laboratory of Alzheimer’s Neuroimaging and Epidemiology - LANE, IRCCS Istituto Centro San Giovanni di Dio Fatebenefratelli, Brescia, Italy

**Keywords:** alzheiemer’s disease, patient subtyping, patient staging, SuStain model, inter-cohort validation

## Abstract

Alzheimer’s disease (AD) is a neurodegenerative disorder which spans several years from preclinical manifestations to dementia. In recent years, interest in the application of machine learning (ML) algorithms to personalized medicine has grown considerably, and a major challenge that such models face is the transferability from the research settings to clinical practice. The objective of this work was to demonstrate the transferability of the Subtype and Stage Inference (SuStaIn) model from well-characterized research data set, employed as training set, to independent less-structured and heterogeneous test sets representative of the clinical setting. The training set was composed of MRI data of 1043 subjects from the Alzheimer’s disease Neuroimaging Initiative (ADNI), and the test set was composed of data from 767 subjects from OASIS, Pharma-Cog, and ViTA clinical datasets. Both sets included subjects covering the entire spectrum of AD, and for both sets volumes of relevant brain regions were derived from T1-3D MRI scans processed with Freesurfer v5.3 cross-sectional stream. In order to assess the predictive value of the model, subpopulations of subjects with stable mild cognitive impairment (MCI) and MCIs that progressed to AD dementia (pMCI) were identified in both sets. SuStaIn identified three disease subtypes, of which the most prevalent corresponded to the typical atrophy pattern of AD. The other SuStaIn subtypes exhibited similarities with the previously defined hippocampal sparing and limbic predominant atrophy patterns of AD. Subject subtyping proved to be consistent in time for all cohorts and the staging provided by the model was correlated with cognitive performance. Classification of subjects on the basis of a combination of SuStaIn subtype and stage, mini mental state examination and amyloid-β_1-42_ cerebrospinal fluid concentration was proven to predict conversion from MCI to AD dementia on par with other novel statistical algorithms, with ROC curves that were not statistically different for the training and test sets and with area under curve respectively equal to 0.77 and 0.76. This study proves the transferability of a SuStaIn model for AD from research data to less-structured clinical cohorts, and indicates transferability to the clinical setting.

## Introduction

Interest in the application of advanced statistics and machine learning (ML) in medicine has been constantly rising during the last years and their predictive capability allowed advancements in many fields. Particularly, data-driven approaches may contribute greatly to the advancement of neurosciences (Oxtoby et al., 2017; Ten Kate et al., 2018; Redolfi et al., 2020), where diseases are regularly modeled heuristically and patient care is influenced by clinicians’ expertise (Braak and Braak, 1991; Jack et al., 2010; Jack et al., 2013).

Alzheimer’s disease (AD) is one of the most impactful neurodegenerative diseases, affecting more than 50 million patients worldwide and costing healthcare systems $800 billion per year (Chan et al., 2019). The common underlying pathology of this disease is the combination of deposition of amyloid plaques with tau neurofibrillary tangles (NFT) (Braak and Braak, 1991), which is the driving cause of neurodegeneration and brain atrophy that leads to a progressive cognitive deterioration that affects multiple domains and eventually to a complete loss of function (Jack et al., 2010). Some basic questions still remain unresolved, such as: how homogeneous is AD? Is the course of progression more or less the same for most patients or are there significant variations?

Heuristic models of the temporal evolution of AD have been largely hypothesized (Braak and Braak, 1991; Jack et al., 2010; Jack et al., 2013), but most of these had the limitation of defining a mean average for the disease evolution that fits the majority of the AD patients. Instead, the phenomenology of AD is heterogeneous in terms of spatial distribution of tau NFT (Murray et al., 2011) and detecting rarer disease patterns may help in patient stratification, potentially allowing for specific drug targeting (ten Kate et al., 2018). Another major limitation of most heuristic and data driven models is the lack of validation in independent data, which is fundamental in order to translate models from the research setting to the clinical practice. For all these reasons well-validated ML tools are needed in order to promote advancements in clinical practice.

In recent years, the collection of numerous data sets containing demographic, clinical and biologic data of subjects from all stages of AD made possible the employment of statistical models and ML approaches (Oxtoby and Alexander, 2017). This context helped deploying disease models that allowed the definition of new strategies for biomarker-informed patient staging (Sperling et al., 2011). Among these algorithms, the family of event-based models (EBM) has been proven successful in defining discrete models for a wide battery of brain diseases (Young et al., 2015; Eshaghi et al., 2018; Wijeratne et al., 2018; Venkatraghavan et al., 2019; Firth et al., 2020; Oxtoby et al., 2021), showing utility in fine-grained staging of patients (Young et al., 2014). Generally, the assumption of these EBMs is that the sequence of events describing the disease progression is common for all subjects, which ignores the observed variation between individuals that may indicate the presence of subtypes of AD (Poulakis et al., 2020).

One key limitation of early subtyping approaches in literature (Whitwell et al., 2012; Nettiksimmons et al., 2014; Noh et al., 2014; Hwang et al., 2015), is that they do not account for temporal variation of the disease, implicitly assuming that all subjects were at the same disease stage.

SuStaIn (Young et al., 2018) (Subtype and Stage Inference) generalizes the EBM approach to include both subtyping and staging of subjects simultaneously, by using a full trajectory of change to define each subtype rather than a static pathology pattern. SuStaIn drops the basic EBM hypothesis of a single event sequence that fits all subjects, while also modeling the transition of biomarkers between different intermediate levels of severity rather than just changing from normal to abnormal. SuStaIn enables the discovery of different progression patterns that represent different manifestations of the same disease while avoiding the confounds of temporal change (Young et al., 2018).

However, SuStaIn has been tested so far only on well-defined research datasets or on synthetic data. Well-defined research datasets are not entirely representative of the general population (Ferreira et al., 2017) and transferability of a model to a less-structured clinical data is not granted a priori. In this paper we trained SuStaIn model on the well-defined research dataset of Alzheimer’s disease Neuroimaging Initiative (ADNI) (Aisen et al., 2010), and we tested the subtyping and staging utility provided by the resulting disease model on a wider and heterogeneous data cohort composed of independent and less-well-phenotyped datasets representative of clinical settings and routine biomarker collection procedures. Our goal was to assess the transferability of a SuStaIn progression model from research data to an independent clinical data cohort coming from three different multi-centric data sets encompassing the entire AD spectrum that spans from early pre-clinical stages of cognitively normal (CN) elderly individuals to full blown dementia. This is a mandatory step in order to adopt SuStaIn and, more generally, advanced statistical models and ML tools in the clinical environment.

## Materials and Methods

### Participants

Data from a total of 1810 subjects gathered from various cohorts (Table 1) were used for this study. Subjects were divided into a training set, used to create the disease model, and a test set, used for model validation. The training set was composed of baseline data of 1043 subjects from the ADNI cohort that were either CN, affected by mild cognitive impairment (MCI) or AD dementia (Table 2), and were not affected by other major neurological diseases. Subjects diagnosed with subjective memory complaints (SMC) were included in the CN group since Mini-Mental State Examination (MMSE) score of these individuals was 28.1 ± 1.6. Diagnostic criteria used to identify MCI subjects were a clinical dementia rating (CDR) = 0.5 and a mini mental state examination (MMSE) (Tombaugh and McIntyre, 1992) score ≥24, while AD subjects were identified as all subjects with CDR ≥ 1 or subjects with CDR = 0.5 and MMSE<24.

**TABLE 1:** Characteristics of the data sets selected.

	Data Set	Full name	Description	Categories
Training Set	ADNI-1	Alzheimer’s Disease Neuroimaging Initiative – 1	The Alzheimer’s Disease Neuroimaging Initiative Aisen et al. (2010) is a longitudinal multicentre study designed to develop clinical, imaging, genetic, and biochemical biomarkers for the early detection and tracking of Alzheimer’s disease (AD). ADNI was originally launched in 2003 as a public-private partnership; its primary goal has been to test whether magnetic resonance imaging (MRI), biological markers, clinical and neuropsychological assessments can be combined to measure the progression of MCI and Alzheimer’s disease. The initial five-year study (ADNI-1) was extended by 2 years in 2009 by a Grand Opportunities grant (ADNI-GO), and in 2011 by further competitive renewal of the ADNI-1 grant (ADNI-2). Through its three phases, it has targeted participants with AD, different stages of MCI, and CN.	CN MCI
				AD
				SMC
	ADNI-GO	Alzheimer’s Disease Neuroimaging Initiative – Grand Opportunities		MCI
				SMC
	ADNI-2	Alzheimer’s Disease Neuroimaging Initiative – 2		CN
				MCI
				AD
				SMC
Test Set	OASIS	Open Access Series of Imaging Studies	OASIS Marcus et al. (2007) consists of I) a cross-sectional collection of 416 subjects. 100 of the included subjects, over the age of 60, have been clinically diagnosed with very mild to moderate Alzheimer’s disease (AD). II) A longitudinal collection of 150 subjects aged from 60 to 96 years. Each subject was scanned on two or more visits, separated by at least 1 year for a total of 373 imaging sessions. In addition, the data set contains socio-demographic, clinical, and genotype information.	CN
				MCI
				AD
	PharmaCog (E-ADNI)	Prediction of cognitive properties of new drug candidates for neurodegenerative diseases in early clinical development	PharmaCog is an industry-academic (Innovative Medicines Initiative – IMI) European project aimed at identifying biomarkers sensitive to symptomatic and disease modifying effects of drugs for Alzheimer’s disease Galluzzi et al. (2016). Several clinical sites participated in this study across Italy (Brescia, Verona, Milan, Perugia, and Genoa), Spain (Barcelona), France (Marseille, Lille, and Toulouse), Germany (Leipzig and Essen), Greece (Thessaloniki) and Netherland (Amsterdam). 151 MCI patients have been studied longitudinally for 3 years collecting multimodal image scans, clinical variables, and bio-specimens.	MCI
				AD
	ViTA	Vienna Transdanube Aging	ViTA is a population-based cohort-study of all 75-years old inhabitants of a geographically defined area of Vienna Fischer et al. (2002). VITA is composed of 606 subjects followed longitudinally for 4 years. Recruitment took place between May 2000 and October 2002. The primary focus of the VITA work-group was to establish a prospective age cohort for evaluation of prognostic criteria for the development of AD.	CN
				MCI
				AD

AD, Alzheimer’s disease; CN, cognitively normal; MCI, mild cognitive impairment; SMC; subjective memory complaints.

**TABLE 2:** Demographic, clinical, genetic and biological characteristics of the training and test sets.

		N	Age (years)	Sex (M/F)	Education (years)	MMSE (raw score)	Aβ_1-42_ (positive/negative)	*APOE*-ε4 (carriers/ non carriers)
Training set	CN	335	73.5 ± 5.9	46%/54%	16.3 ± 2.6	29.1 ± 1.2	40%/60%	27%/73%
	MCI	537	72.0 ± 7.2	59%/41%	16.0 ± 2.8	27.7 ± 1.8	66%/34%	51%/49%
	AD	171	73.4 ± 8.2	54%/46%	15.5 ± 2.7	23.4 ± 2.0	95%/5%	73%/27%
	Total	1043	72.7 ± 7.0	54%/46%	16.04 ± 2.7	27.4 ± 2.5	62%/38%	46%/54%
	sMCI	271	72.3 ± 7.1	58%/42%	16.1 ± 2.8	28.0 ± 1.7	56%/44%	42%/58%
	pMCI	205	73.1 ± 6.8	59%/41%	15.8 ± 2.8	27.2 ± 1.8	87%/13%	64%/36%
Test Set	CN	440	54 ± 25*	37%/63%*	8.4 ± 5.7*	29.0 ± 1.2	NA	2%/7%
	MCI	283	72.3 ± 7.6	46%/54*	9.2 ± 5.1*	26.3 ± 2.6*	34%/17%	19%/32%*
	AD	44	77.3 ± 7.4*	34%/66%*	5.8 ± 5.3*	21.7 ± 3.8*	NA	0%/5%
	Total	767	62 ± 21*	40%/60%*	8.6 ± 5.4*	27.2 ± 3.0	12%/6%	8%/16%*
	sMCI	152	71.2 ± 7.5	47%/53%	11.5 ± 4.2*	26.7 ± 2.2*	46%/25%	25%/43%
	pMCI	39	69.8 ± 6.4*	49%/51%	11.7 ± 3.9*	25.7 ± 2.4*	44%/5%	33%/26%

Values from CN, MCI and AD contribute to the totals, MCI subpopulations of pMCIs and sMCIs are reported as well. Values marked with * on the test set are significantly different (*p*-value of ANOVA for continuous variables and chi-square for discrete variables <0.05) from the corresponding values from training set. Abbreviations: M, male; F, female; N, number.

Additionally, two subpopulations of subjects with longitudinal information, namely stable MCI subjects (sMCI) and progressive MCI (pMCI) were identified. Specifically, sMCIs were subjects for which only MCI diagnosis was reported for all available time-points and pMCIs were subjects that had at least one diagnosis of MCI and subsequently one diagnosis of AD and never reverted to MCI in the time-span of 10 years we considered.

The test set was composed of subjects coming from three independent data cohorts characterized by heterogenous and less-structured data collection. Specifically, subjects were selected from the Open Access Series of Imaging Studies (OASIS) (Marcus et al., 2007), PharmaCog (Galluzzi et al., 2016), and Vienna Transdanube Aging (ViTA) (Fischer et al., 2002) cohorts, totaling 767 subjects with the same clinical labels and diagnostic criteria as the training set. Populations of sMCIs and pMCIs were identified in the test sets with the same criteria as in the training set, but in this case the maximum time-span available was 7.5 years.

The training and test set populations were heterogeneous in terms of demographic, genetic and biological features (Table 2). The CN subjects in the test set were younger and less educated compared to the training set. The MCI subjects in the test set were less educated, and had higher prevalence of *APOE*-ε4 non-carriers compared to the training set’s. Moreover, the pMCIs in the test set were younger than those in the training set. Finally, the AD dementia subjects in the test set were older and less educated compared to the corresponding subjects of the training set. Importantly, no statistical differences were reported in the frequency of abnormal cerebrospinal fluid (CSF) concentrations of amyloid-β_1-42_ (Aβ_1-42_) protein between the test and the training sets for each diagnostic group. In all test set subgroups, with the exception of pMCIs, the gender prevalence was statistically different compared to the training set.

### Clinical, Cognitive, Biological and Imaging Data

Clinical, cognitive, biological and imaging information were collected for each subject from the training and test set. Imaging information was derived from 1.5T or 3T T1-3D magnetic resonance imaging (MRI) scans, and was analyzed with Freesurfer 5.3 cross sectional stream (http://surfer.nmr.mgh.harvard.edu) with Desikan-Killiany atlas to obtain volumes of relevant brain regions of each subject, which were used to build the SuStaIn disease progression model. Freesurfer outputs were visually checked and validated by expert neuroscientists. The volumes of specific regions were used, specifically, we selected volumes of hippocampus, fusiform gyrus, entorhinal cortex, middle temporal cortex, precuneus, amygdala, insula, thalamus putamen, caudate, nucleus accumbens, pallidum and ventricles, which are among the most used regions employed in both heuristic and data driven currently available atrophy models for AD (Frisoni et al., 2010; Vemuri and Jack, 2010; Koval et al., 2018; Young et al., 2018; Archetti et al., 2019). For each region, volumes were obtained averaging the respective volume of the left and right hemisphere, volume of ventricles was obtained as the sum of 3rd and lateral ventricles. Cognitive information was provided by the MMSE score and was used as a proxy in order to verify that the disease model correlated with cognitive decline. Biological data included CSF concentration of Aβ_1-42_ protein and it was used to identify a subpopulation of amyloid-negative healthy subjects defined as those CN subjects from the training set that had an Aβ_1-42_ CSF concentration >192 pg/ml (Shaw et al., 2009). For the training set, Aβ_1-42_ CSF concentration was obtained with Multiplex xMAP Luminex platform with Innogenetic immunoassay kit–based reagents (Kang et al., 2012). For demographic purposes Aβ_1-42_ CSF concentration was collected for the test set subjects as well, but the CSF biomarker was only available for PharmaCog subjects. In this case, Aβ_1-42_ CSF concentration was obtained with Enzyme Linked Immunosorbent Assay (ELISA) (Butler, 2000) which led to different CSF biomarkers distributions with respect to the training set. In order to tackle this issue, Aβ_1-42_ CSF concentrations from PharmaCog were rescaled to match the mean and standard deviation of Aβ_1-42_ distribution of training set subjects. The same cut-off value as the training set was used to define abnormality. As a compensation for inter-cohort demographic variability all volumetric measures for both training and test sets were corrected against the effect of age, sex, education (Gale et al., 2007), *APOE* genotype (Liu et al., 2013) and total intracranial volume (TIV) (Gur et al., 1991; Király et al., 2016) by means of multiple linear regression, and were converted into z-scores with respect to the mean and standard deviation defined by the volumes distribution of the healthy amyloid-negative subjects from the training set. Correction of biomarkers was performed separately for training set and test set.

### Modelling

The disease progression model was built using the SuStaIn algorithm (Young et al., 2018), which generalizes the EBM approach (Fonteijn et al., 2012; Young et al., 2015) to allow for subtyping. Traditional EBMs rely on the assumption that it is possible to define a common sequence of events where, in the case of disease models, each event is defined as the value of a biomarker stepping from normality to abnormality. The normality and abnormality of the values are usually defined on the basis of biomarker distributions of healthy and diseased subjects. However, SuStaIn differs from classical EBM models in two main features:

1) The hypothesis of the common event sequence is relaxed in favor of multiple event sequences corresponding to a data-driven number of different disease subtypes that represent different disease trajectories of biomarker change observed in the training set. The optimal number of subtypes is determined using a popular model selection criterion called “Cross Validation Information Criterion” (CVIC) (Gelman et al., 2014).
2) Biomarkers are not treated as binary entities that are either normal or abnormal but all biomarker trajectories are modeled as a succession of z-scores progressing linearly toward abnormality.

Considering such modifications, the disease progression model is then represented by a set of sequences of integer z-scores for each biomarker, which represents the different disease subtypes. For this work z-scores were calculated with respect to the mean and standard deviation defined by the biomarker distribution of the healthy amyloid-negative ADNI subjects.

The maximum number of subtypes was set to 5 and the maximum value of z-scores for each biomarker was set to 3 (Young et al., 2018), meaning that maximum abnormality of each biomarker was reached when the z-score was >= 3.

When the disease progression model is defined, it is possible to outline the subtype that most likely fits any subject as the subtype for which the likelihood of a subject’s z-scores projected on the z-score progression is maximized (Young et al., 2018). The subject is then staged on the most likely stage of the z-score progression defined by his or her subtype. The SuStaIn algorithm is publicly available in the form of a python package at the following link: http://europond.eu/software/.

### Model Validation and Statistical Analysis

In order to investigate possible similarities with other subtyping methods, correlation between subtypes defined with SuStaIn and subtypes defined on the basis of visual rating scales of regional brain atrophy (Ferreira et al., 2019) was explored. Specifically, the visual scales considered were Scheltens’ medial temporal atrophy (MTA) scale (Scheltens et al., 1992), Koedam’s scale for Posterior Atrophy (PA) (Koedam et al., 2011) and Pasquier’s frontal subscale of global cortical atrophy (GCA-F) (Pasquier et al., 1996; Scheltens et al., 1997).

According to visual ratings, typical AD was defined as abnormal MTA together with abnormal PA and/or abnormal GCA-F. Hippocampal-sparing was characterized by abnormal PA and/or abnormal GCA-F but normal MTA, while minimal atrophy AD was defined as normal scores in MTA, PA, and GCA-F. Limbic-predominant was defined as abnormal MTA alone with normal PA and GCA-F (Ferreira et al., 2017). All the visual ratings were computed automatically by means of the Automatic Visual Ratings of Atrophy (AVRA) tool (Mårtensson et al., 2019).

Further heuristic validation of SuStaIn was tested by exploring correlation of the subjects staging to the cognitive decline measured by means of MMSE.

The transferability of the model to new individuals was tested by subtyping and staging subjects from both the training and test sets on the basis of baseline volumes. Similarities between clinical, demographic, genetic and CSF features of subjects from the training and test sets assigned to different subtypes were explored by means of ANOVA and chi-square tests.

A subset of subjects (502 for the training set and 139 for the test set) were subtyped using 12-months visit biomarkers measurement in order to check the temporal consistency of the subtyping. Predictive capabilities of the model were tested by measuring the area under curve (AUC) of receiver-operator characteristic (ROC) curves obtained from classification of pMCIs and sMCIs from the training and test sets using various combinations of subtype, stage, MMSE and CSF Aβ_1-42_ concentration as predictors in a multivariate logistic model. Statistical differences between ROC curves were tested by means of De Long test (DeLong et al., 1988). All ROC analyses were computed using R (version 3.5.1).

Chi-square and ANOVA tests (*α* = 0.05) were performed in python (version 3.6.9) to test differences between the diagnostic groups and subtypes.

## Results

**FIGURE 1:**
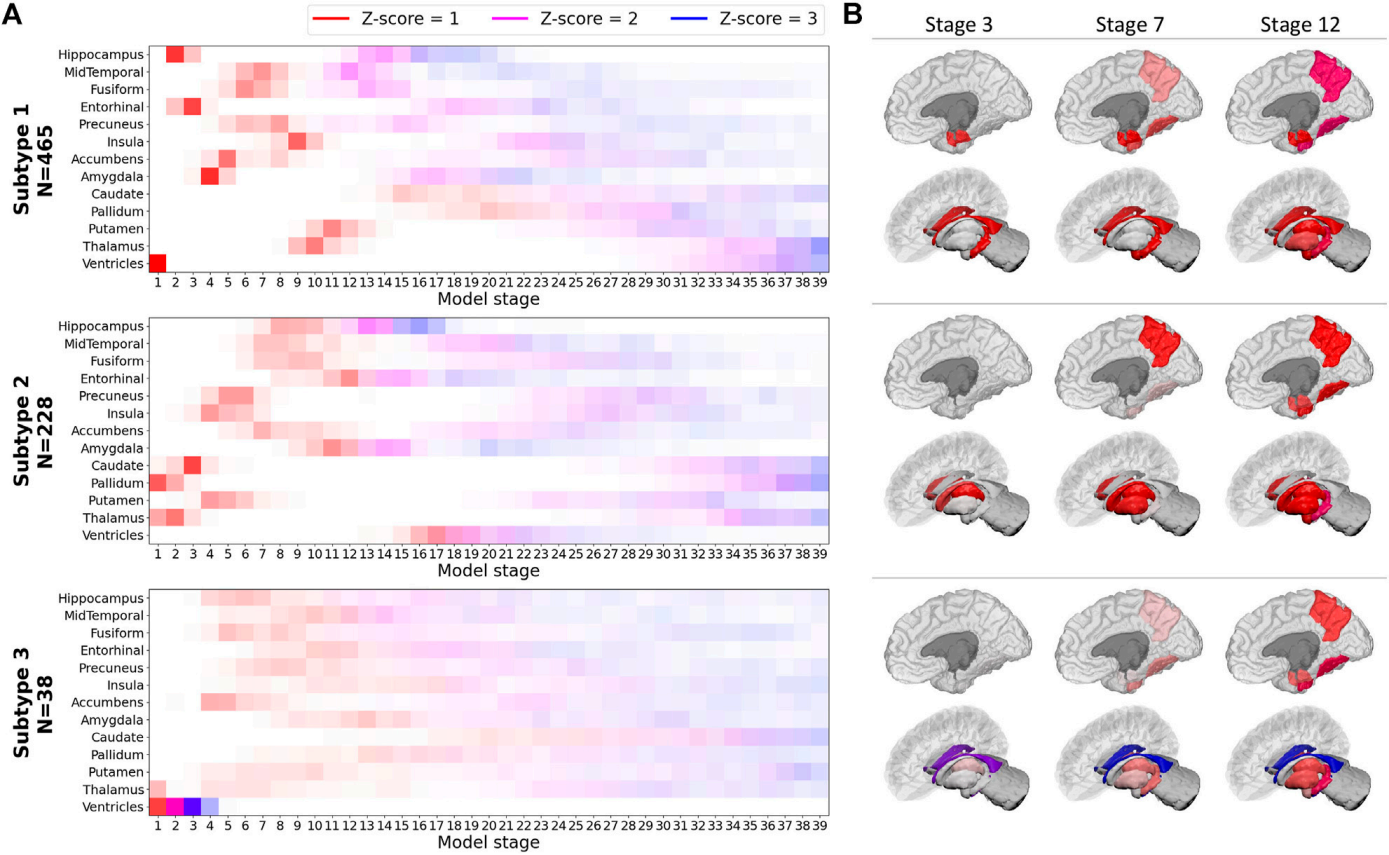
SuStaIn model built on the basis of volumetric biomarkers of the training set.**(A)** Z-score progression patterns for each subtype. Color shades indicate the probability of a Z-score to increment, “N” indicates the number of subjects from the training set assigned to each subtype **(B)** Representations of early stages for each subtype.

The disease model identified by SuStaIn consisted of three disease subtypes (Figure 1). The first disease subtype (“Subtype 1” in the next sections), is characterized by abnormality (Z-score = 1) that can be observed in the ventricles first, then atrophy occurs in the hippocampus and entorhinal cortex, that are also the first regions to show full abnormality (Z-score = 3) alongside amygdala. Interestingly, ventricles are also the last regions to show full abnormality meaning a relatively slow but persistent volumetric expansion process that tracks the disease progression.

The second disease subtype (“Subtype 2” in the next sections) shows an atrophy pattern where abnormality starts in thalamus and pallidum (Z-score = 1). Subsequently, atrophy can be observed in caudate, putamen, insula, precuneus and then fusiform gyrus and middle-temporal cortex and hippocampus which is the first biomarker to become fully abnormal (Z-score = 3). In this subtype, ventricles start expanding later than in Subtype 1. The third subtype (“Subtype 3” in the next sections) shows an atrophy pattern where ventricles become fully abnormal before atrophy starts in almost all the other regions, for which a less-defined atrophic progression is manifested in comparison to Subtypes 1 and 2.

**FIGURE 2:**
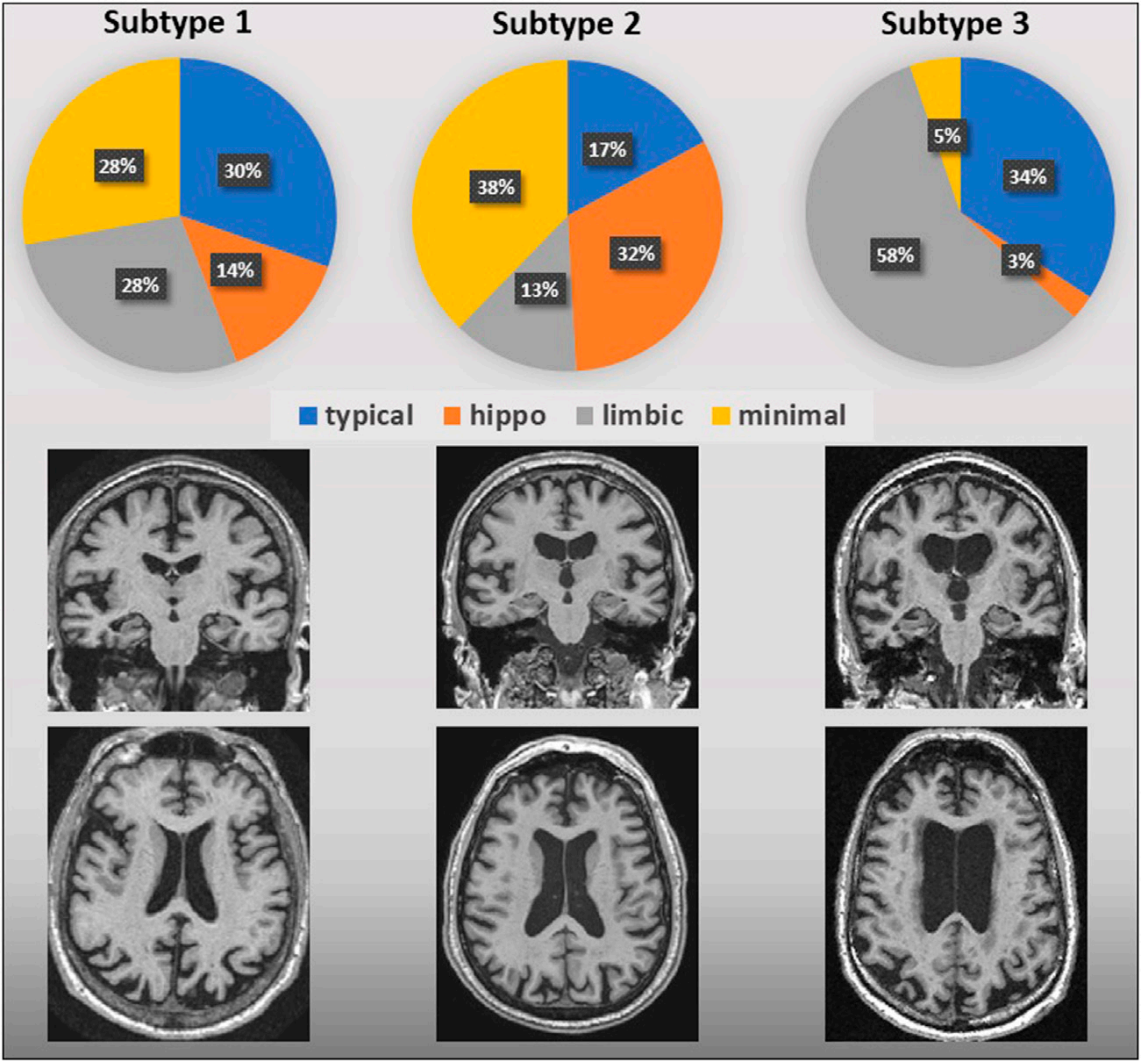
AVRA vs. SuStaIn subtypes of AD. Pie graphs represent the percentage of AVRA subtypes subjects for each SuStaIn subtype. Regional atrophy in AVRA was measured with the MTA, PA and GCA-F scales based on T1-3D weighted images; below, visual examples of the SuStaIn atrophy subtypes are shown.

**FIGURE 3:**
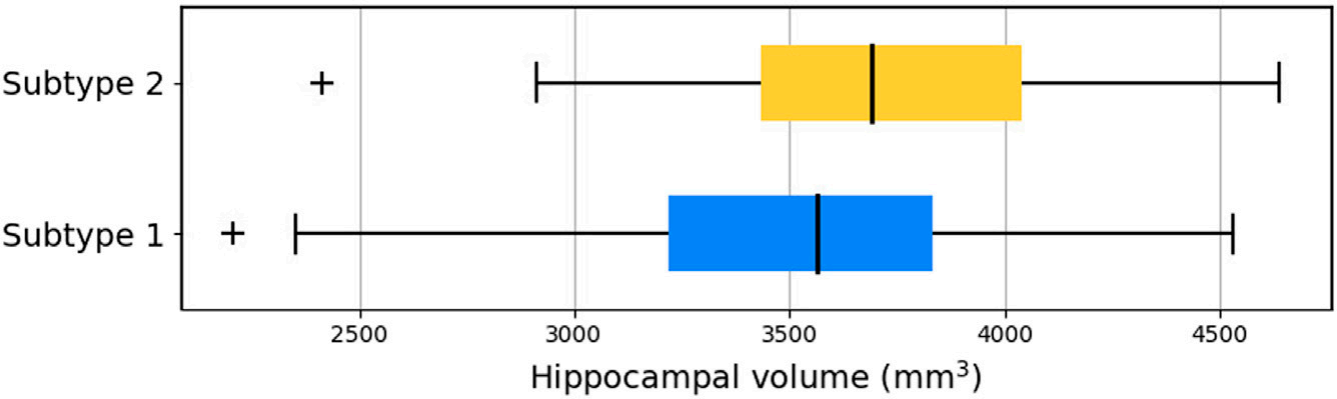
Hippocampal volume of subjects from Subtypes 1 and 2 labeled with minimal atrophy according to AVRA scores. Hippocampal volumes were averaged between right and left hemisphere for simpler representation.

SuStaIn subtypes were cross linked to AVRA ratings to evaluate whether similarities between subtypes defined by the two methods exist (Figure 2). Subtype 1 was mainly characterized by the “Typical AD” atrophy pattern (Ferreira et al., 2019); Subtype 2 showed an equal predominance of the hippocampal-sparing variant; Subtype 3 showed a limbic-predominant subtype. The minimal atrophy subtype (Ferreira et al., 2020) was most consistent with Subtypes 1 and 2. After correcting against effects of sex, age and TIV, relevant differences (p-value for ANOVA <0.05) in volume of hippocampus were observed between subjects from Subtypes 1 and 2 labeled with minimal atrophy according to the AVRA scores (Figure 3), with subjects from Subtype 2 exhibiting larger volumes. Subjects with minimal atrophy from Subtype 3 are not reported as they are not enough for statistical significance.

Differences in AVRA visual scores between subtypes were inferred *via* a linear regression model of visual scores vs. model stage (Supplementary Figure S1). No relevant subtype differences were observed for GCA. MTA was shown to progress significantly faster for Subtype 2 than Subtypes 1 and 3. Subtype 3 also showed a significantly faster progression of the PA scale. Subjects from each diagnostic category of both training and test sets that were assigned to a specific subtype are shown in Table 3. Subjects that were in stage 0 or in the final stage were excluded from the subtyping as these stages are equivalent for each subtype. In each diagnostic group, the majority of subjects were on average assigned to the typical subtype (65% for training set and 82% for the testing set). A minority of the subjects were assigned to the hippocampal sparing subtype, specifically 30% of the training set and 16% for the test set, while only a limited number of subjects for each dataset were assigned to the limbic subtype (5% for the training set and 2% for the test set). For both sets, subjects from each diagnostic category were staged on average at stages that mirror the worsening of their clinical condition (Table 3), with the exception of pMCIs and sMCIs from Subtype 3.

**TABLE 3:** Number and percentage of subjects from each diagnostic category assigned to each subtype.

		Subtype 1		Subtype 2		Subtype 3	
		N	Average Stage	N	Average Stage	N	Average Stage
Training Set	CN	96 (54%)	3 ± 3	74 (41%)	3 ± 3	9 (5%)	4 ± 1
	MCI	243 (62%)	5 ± 4	128 (33%)	5 ± 5	22 (5%)	7 ± 4
	AD	126 (79%)	8 ± 5	26 (16%)	9 ± 6	7 (5%)	11 ± 5
	sMCI	111 (59%)	4 ± 4	67 (36%)	4 ± 4	10 (5%)	8 ± 5
	pMCI	116 (69%)	6 ± 5	44 (26%)	7 ± 6	8 (5%)	7 ± 4
Test Set	CN	303 (86%)	5 ± 4	37 (11%)	5 ± 4	9 (3%)	5 ± 2
	MCI	185 (78%)	7 ± 6	49 (21%)	6 ± 4	3 (1%)	9 ± 2
	AD	41 (95%)	9 ± 7	1 (2.5%)	12	1 (2.5%)	9
	sMCI	83 (68%)	7 ± 6	35 (29%)	5 ± 4	4 (3%)	8 ± 3
	pMCI	32 (84%)	9 ± 6	6 (16%)	11 ± 4	0 (0%)	NA

Significant differences between subtypes were observed for demographic, clinical, biological and genetic variables (Table 4). For each subtype, subjects from all diagnostic categories were considered. In both training and test sets, subjects from Subtype 2 were on average more educated and a larger portion of them were male with respect to subjects from Subtype 1. Similarly, subjects from Subtype 3 had a lower MMSE with respect to Subtype 2. In the training set, where CSF data was widely available, the portion of subjects that had an abnormal Aβ_1-42_ CSF concentration was significantly lower with respect to the other subtypes. This effect was not observed in the test set for the small number of subjects for which Aβ_1-42_ is available.

**TABLE 4:** Descriptive statistics of the demographic, clinical, biological and genetic variables of subjects for each subtype

		Age (years)	Sex (M/F)	Education (years)	MMSE (raw score)	Aβ_1-42_ (positive/negative)	*APOE*-ε4 (carriers/ non carriers)
Training Set	Subtype 1	72.5 ± 7.2^a^	48%/52%^a^	15.9 ± 2.7^a^	26.6 ± 2.6^a^	72%/28%^a^	48%/52%
	Subtype 2	73.8 ± 6.7^a^	84%/16%^a,b^	16.4 ± 2.7^a^	27.9 ± 2.0^a,b^	53%/46%^a,b^	44%/56%
	Subtype 3	74.8 ± 6.2	61%/39%^b^	15.9 ± 3.0	26.5 ± 2.6^b^	79%/21^b^	42%/58%
Test Set	Subtype 1	60 ± 24^c^	41%/59%^a^	8.5 ± 5.4^a^	26.7 ± 3.3	3%/10%	8%/14%
	Subtype 2	63 ± 17^b^	64%/36%^a^	10.4 ± 5.6^a^	27.4 ± 2.2^b^	31%/15%	24%/25%
	Subtype 3	74.4 ± 5.7^c,b^	46%/54%	7.9 ± 6.1	25.7 ± 4.7^b^	15%/0%	0%/15%

Values marked with ^a^indicate significant differences (*p*-value < 0.05) between Subtype 1 and Subtype 2 values in the same set; values marked with ^c^indicate significant differences (*p*-value < 0.05) between Subtype 1 and Subtype 3 values in the same set; values marked with ^b^indicate significant differences (*p*-value < 0.05) between Subtype 2 and Subtype 3 values in the same set.

**FIGURE 4:**
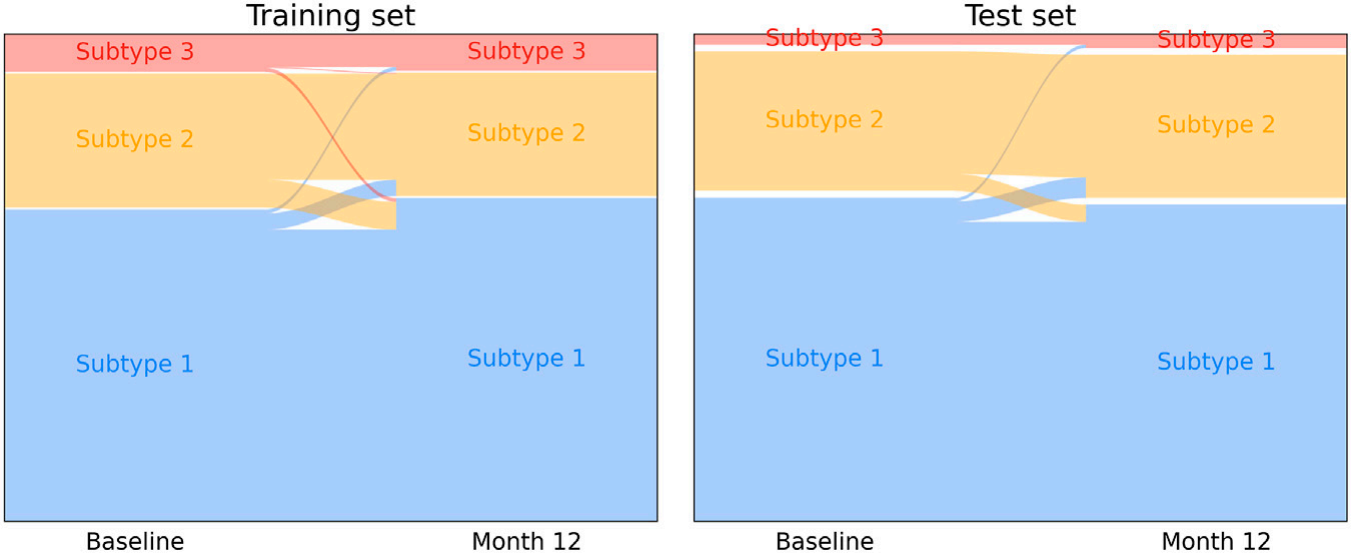
Longitudinal subtype consistency for training set subjects **(left)** and test set subjects **(right)** over a 12-months follow-up period.

Subtyping consistency of the SuStaIn progression model was tested by comparing subtyping of subjects for which 12-months follow-up was available (502 for the training test and 140 for the test set). Few subjects were subtyped to a different group at 12-months follow up (Figure 4), with only 11% of training set subjects and 9% of test set subjects assigned to different subtypes. Changes occurred mainly between subtypes 1 and 2 in both training and test sets. For subjects with stable subtype assignment, stage progression was relatively slow in time showing an average progression of 0.8 ± 1.5 stages over the 12-month period.

**FIGURE 5:**
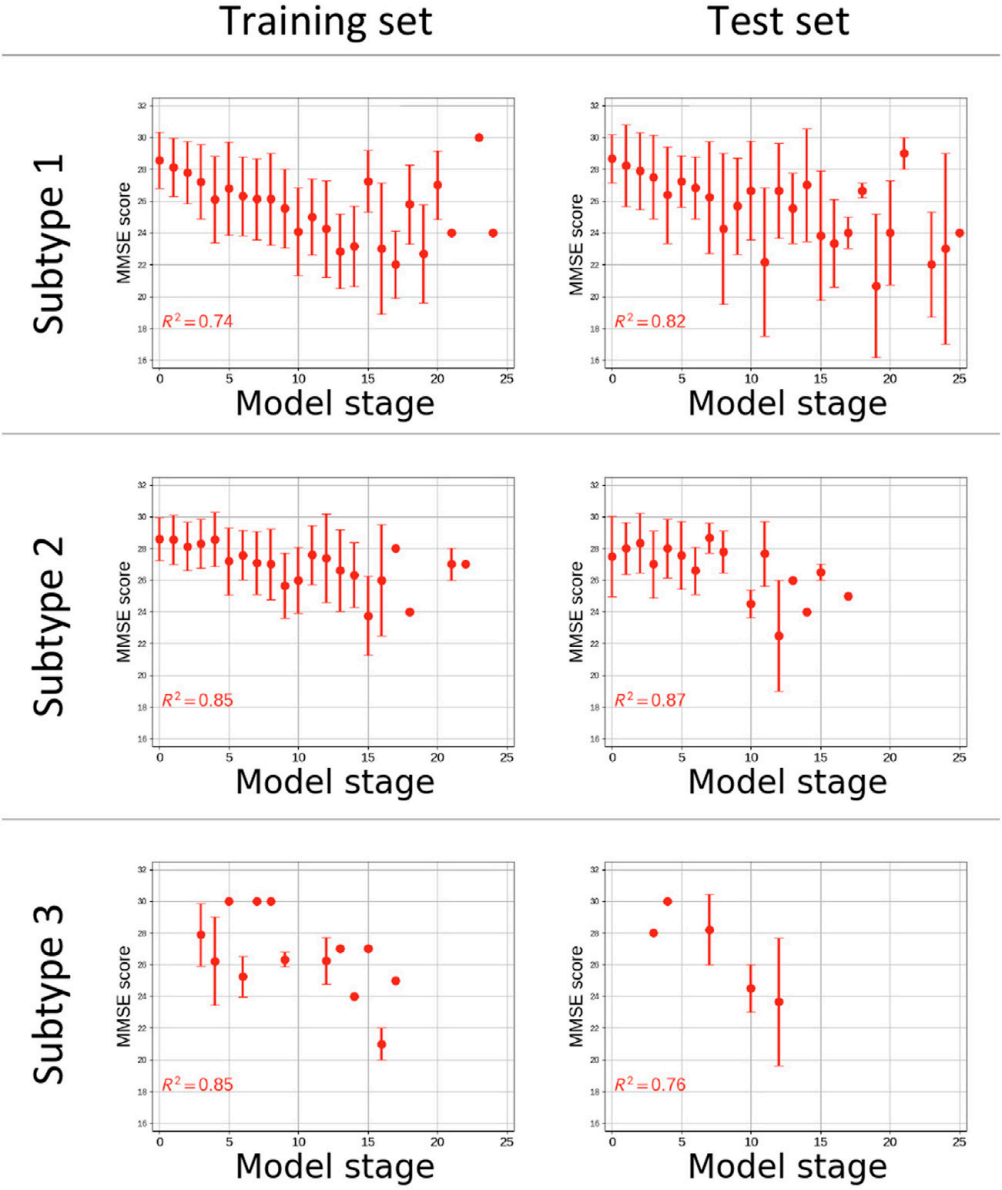
Plot of Cognitive performance measured by Mini Mental State Examination (MMSE) vs. the estimated disease stage subjects from the training **(left)** and test **(right)** sets for each subgroup. Coefficients of determination (R^2^) of the linear regression of MMSE score vs. disease stage are reported. The *x*-axes are only reported up to stage 25 of 39 as no subjects were staged beyond.

The disease progression signature defined by Subtype 1 showed good correlation with cognitive performance measured by MMSE (Figure 5), with R^2^ = 0.74 for the training set and R^2^ = 0.82 for the test set. Similarly, good correlations were registered in Subtype 2 (R^2^ = 0.85 training set; R^2^ = 0.87 test set) and Subtype 3 (R^2^ = 0.85 training set; R^2^ = 0.76 test set).

Classification of pMCIs and sMCIs, based on subtype and stage retuned ROCs with AUC = 0.67 for the training set and 0.72 for the test set. The combination of subtype and stage with other predictors tracking different aspects of the disease, namely the MMSE and CSF concentration of Aβ_1-42_ protein, returned a better classification performance than the subtype and stage model alone, with AUC = 0.77 for the training set and AUC = 0.76 for the test set, outperforming also a model that accounts only for MMSE and Aβ_1-42_ (AUC = 0.72 for the training set and AUC = 0.74 for the test set) and a model that accounts for AVRA subtype, MMSE and Aβ_1-42_ (AUC = 0.72 for the training set, unavailable for the test set). Notably, for each predictor combination no statistically significant differences were observed between ROC curves (Supplementary Figure S2) of the training and test sets (p-value of DeLong test >0.05).

## Discussion

In this study, we tested the transferability of a SuStaIn AD progression model among clinical data cohorts. The disease progression model trained on volumetric imaging markers from an observational research study estimated three AD-related atrophy patterns. Previously, SuStaIn was only tested on research datasets, such as ADNI and GENetic Frontotemporal dementia Initiative (GENFI) or synthetic data (Young et al., 2018), while in the present study we demonstrated model transferability to clinical cohorts through stable and consistent subtyping.

Subtype 1 mirrored the typical course of AD as supposed in heuristic models and as found in previous EBM and data-driven models (Young et al., 2015; Archetti et al., 2019; Venkatraghavan et al., 2019), according to which hippocampus is one of the earliest regions to show considerable atrophy. This subtype also shares similarities with the typical subtype as defined in the original SuStaIn work (Young et al., 2018) for which hippocampus and amygdala are among the first regions to show atrophy. The correspondence of Subtype 1 with the canonical and most prevalent manifestation of AD (Braak and Braak, 1991), is reinforced by our subject subtyping results, with the majority of subjects assigned to this subtype in both training and test set. In particular, the proportions of AD subjects of the training and testing set assigned to Subtype 1, 79% and 95% respectively, are greater than those from other diagnostic categories. Subtype 1 is also majorly prevalent as assignment of pMCIs, with a proportion of 69% compared to the other diagnostic categories.

Subtype 2 shows similarities with the hippocampal-sparing variant of AD characterized by a relative sparing of the medial temporal lobe as observed in previous works (Murray et al., 2011; Whitwell et al., 2012; Ferreira et al., 2019; Krajcovicova et al., 2019). In this subtype hippocampus starts becoming abnormal after most of the others deep gray matter structures, with loss predominantly focused in the insula, caudate nucleus and parietal cortex. The similarity also extends to the demographic characteristics of this group, that is characterized by a higher prevalence of male subjects as reported in previous works (Ferreira et al., 2020). In this subtype, pallidum, putamen and caudate are among the first regions to show atrophy as observed in the subcortical subtype defined in the original SuStaIn work (Young et al., 2018).

Subtype 3 is characterized by a broader atrophy signature with less distinct ordering than the other subtypes, with the exception of ventricles expansion that was clearly the first marker to become abnormal. In this atypical subtype, atrophy seems to progress simultaneously in most brain regions. Subtype 3 was observed in a minority of subjects when considering our whole cohort. These subjects exhibit similarities with the limbic predominant subtype of AD (Ferreira et al., 2017). Also, Subtype 3 might have some characteristics in common with other subtypes as some subjects had been labeled as belonging to the typical AD subtype (Ferreira et al., 2017; Persson et al., 2017). Alternatively, it is possible that this group does not reflect a distinct AD subtype but just includes a subgroup of subjects whose ventricles outlie the normal distribution of ventricles in healthy subjects.

The atrophy subtypes of AD have been assessed *via* visual rating scales in several previous studies (Ferreira et al., 2020). AVRA is a method to automatically quantify these visual rating scales, which was used just on ADNI data, therefore it represented the ideal tool to find a correlate between a clinically used subtyping method and the SuStaIn data driven definition performed on our training dataset. We have produced the first comparison of data-driven subtyping results using a disease progression model (SuStaIn) with existing progression-ignorant methods of visual ratings and AVRA. Partial agreement was observed between SuStain and AVRA subtypes on an individual level, and differences may be imputed to the selection of brain regions used to train SuStaIn, that do not cover entirely the same brain region used to assess visual ratings and to a general lack of harmonization of subtyping methods (Mohanty et al., 2020). SuStaIn proved to offer a finer-grained representation of different atrophy patterns as relevant differences in hippocampal volume were observed between subjects from subtypes 1 and 2 that were labeled with minimal atrophy according to the AVRA scores.

The temporal consistency of SuStaIn subtyping was tested on subjects from the training and test sets for which a 12-months follow-up visit was available. The test resulted in excellent consistency with only 10% of subjects receiving a different subtype assignment across different visits. Since disease stage was relatively stable across the 12-months interval for individuals with stable subtype, the excellent subtype consistency was expected.

Once subjects from all subtypes were staged on the respective disease progression sequence, the SuStain stage showed good linear correlation (Perneczky et al., 2006) with general cognitive decline on the MMSE (Tombaugh and McIntyre, 1992) test, particularly for Subtypes 1 and 2, and the ceiling effect that was observed in previous studies (Hoops et al., 2009; Archetti et al., 2019) was not detected, likely due to the absence of early markers of AD in the model, such as CSF markers.

SuStaIn subtype and stage predicted conversion of MCI subjects to AD with an AUC comparable to other novel statistical algorithms (Ramírez et al., 2018; Salvatore et al., 2018). The combination of multiple predictors proved to be key in improving classification performance as classification based on subtype and stage alone or on MMSE and Aβ_1-42_ alone yielded a lower classification performance. Importantly, classification task performed similarly in the training and test set for each combination of predictors, thus giving a first indication of the transferability of SuStaIn disease models and its use in deep patient phenotypization for future clinical trials as well.

The interpretation of the atrophy subtypes still remains an open issue as solid subtyping ground truth in AD is lacking, since heuristic models such as Jack’s (Jack et al., 2010) or Braak’s (Braak and Braak, 1991) are more aimed at defining a common disease trajectory rather than detecting different atrophy patterns. Also, the model presented here differs slightly from the AD model presented in the original SuStaIn work (Young et al., 2018), and this difference is provoked by choice of different brain regions as input data for the two models and partially due to the different purpose of this study.

Previous works based on cross sectional models were able to reach better classification performances across a wide range of neurological diseases (Willette et al., 2014; Archetti et al., 2019), but in all cases the models were built ab initio using multi-modal markers accounting for biological features and cognitive scores, while we used CSF and cognitive data only for post-hoc analyses. In the present study, we chose to exclude CSF measurements and cognitive scores because these markers were available only for a small portion of subjects used as test set.

The most important limitation of the present work is the relatively small number of subjects used to train and test the model. The small number of subjects particularly affects the characterization of rarer subtypes, that cannot be modeled as accurately as common subtypes. Also, the small number of subjects considered to assess the predictive value of the model prevented us from assessing with a usual power level measures of sensitivity and specificity for the classification of pMCIs and sMCIs.

An important limitation of the model is the relatively low AUC reached in the classification of pMCIs vs. sMCIs, indeed the AUC could be improved with the inclusion of CSF and cognitive scores for the model building phase rather than using them for post hoc analyses (Archetti et al., 2019), but those biomarkers were excluded from the model building as they should not be important factors in atrophy subtype identification. Moreover, CSF and cognitive scores are more easily affected by inter-cohort and inter-centre harmonization issues (Costa et al., 2017; Delaby et al., 2020) thus requiring a more thorough model validation. Therefore, MRI-only models are more suitable for near-future implementation of SuStaIn-based models in tools for subtype detection in single case-scenarios.

Another key factor affecting the AUCs is the unavailability of the characterization in amnestic and non-amnestic MCI for the major portion of the subjects. The condition of amnestic MCI is a more typical prodromal stage for AD that could provide better classification performances (Cousins et al., 2020). Also, the use of amnestic MCIs for the training process could indeed generate a more accurate disease model that better depicts the transition phase from MCI to dementia.

Future work will concentrate efforts in modeling subtypes using larger and more diverse cohorts, that will allow for a more precise definition of subtypes and for a finer-grade characterization of subjects belonging to each subgroup. Another key factor for an optimal definition of the subtypes is the selection of brain regions, and future work will investigate the optimal choice to obtain a disease model that is descriptive and informant without being redundant and trying to maximize the individual match between AVRA subtypes and SuStaIn subtypes. SuStaIn is a suitable approach to build disease models that include non-imaging markers, and future work will investigate the possibility of defining AD progression subtypes based on CSF markers and cognitive scores coupled with imaging markers, possibly linking subtypes with demographic genetic and lifestyle factors.

There are ongoing efforts to extend this work toward full clinical translation. This includes implementing SuStaIn progression models in user-friendly interfaces, external independent validation studies, and usability assessments from clinicians, all of which form key components of the EuroPOND (http://europond.eu/) and E-DADS initiatives (https://e-dads.github.io/).

## Conclusions

We have demonstrated that a data-driven subtyping model (Young et al., 2018) of Alzheimer’s disease progression trained on research-quality MRI (ADNI) is transferable to lower-quality clinical data (PharmaCog, OASIS, ViTA). This is an encouraging result motivated by the expectation that, in the near future, healthcare will increasingly adopt data-driven and ML models in daily clinical practice. Indeed, the validation and generalization of such models on independent datasets is a proof of concept required for their translation from research settings to clinical environments. Open questions remain about the biological mechanisms underpinning Alzheimer’s disease subtypes, which will be an important focus of future studies, including ongoing drug-development efforts.

## Data Availability

Publicly available datasets were analyzed in this study. This data can be found here: adni.loni.usc.edu, neugrid2.eu.

## Abbreviations

AD, Alzheimer’s disease; ADNI, Alzheimer’s disease Neuroimaging Initiative; ANOVA, Analysis of Variance; AUC, Area Under Curve; AVRA, Automatic Visual Ratings of Atrophy; Aβ_1-42_, Amyloid-β_1-42_; CDR, Clinical dementia rating; CN, Cognitively Normal; CSF, Cerebrospinal Fluid; CVIC, Cross Validation Information Criterion; EBM, Event Based Model; ELISA, Enzyme Linked Immunosorbent Assay; GCA-F, Global Cortical Atrophy; GENFI, GENetic Frontotemporal dementia Initiative; MCI, Mild Cognitive Impairment; MMSE, Mini Mental State Examination; MTA, Medial Temporal Atrophy; MRI, Magnetic Resonance Imaging; NFT, Neurofibrillary Tangles; OASIS, Open Access Series of Imaging Studies; PA, Posterior Atrophy; pMCI, progressive Mild Cognitive Impairment; ROC, Receiver-Operator Characteristic; SMC, Subjective memory Complaints; sMCI, stable Mild Cognitive Impairment; SuStaIn, Subtype and Stage inference; TIV, Total Intracranial Volume; ViTA, Vienna Transdanube Aging.
