# Supplementary material for: Inter-Cohort Validation of SuStaIn Model for Alzheimer’s Disease
Source: Front Big Data. 2021 May 20;4:661110. doi: 10.3389/fdata.2021.661110 (PMC8173213; doi:10.3389/fdata.2021.661110)

SUPPLEMENTARY MATERIAL

SF1: Subtype atrophy progression over SuStaIn stages

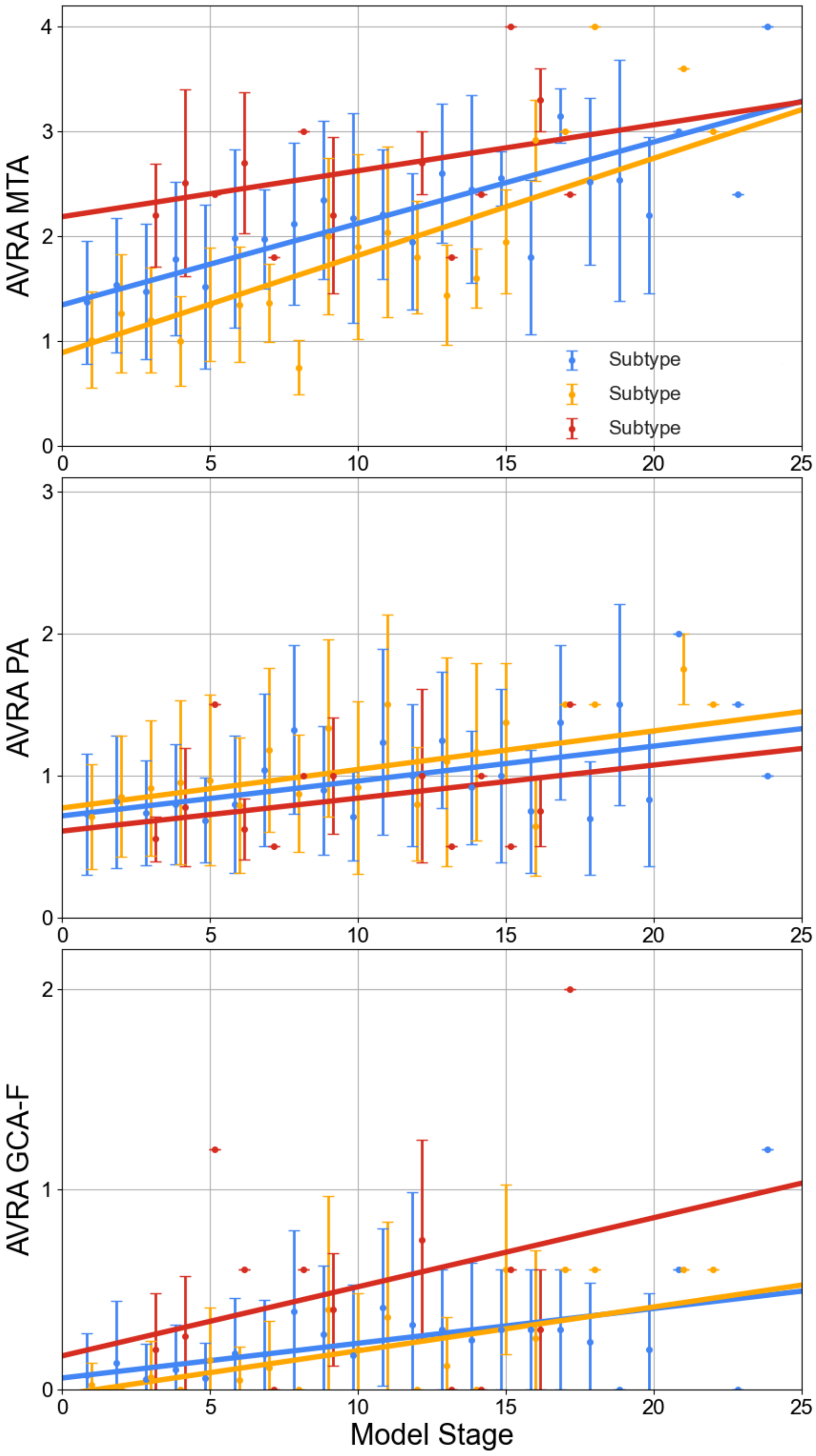

Atrophy progression for the visual rating scales (GCA-F, MTA, PA) over SuStaIn stages. Scores from MTA left and MTA right were averaged between right and left hemisphere for simpler representation

considering progression was similar. Abbreviations: GCA-F: global cortical atrophy – frontal subscale, MTA: Medial Temporal Atrophy, PA: Posterior atrophy.

SF2: ROC curves for classification of pMCIs vs sMCIs form the training and test sets.

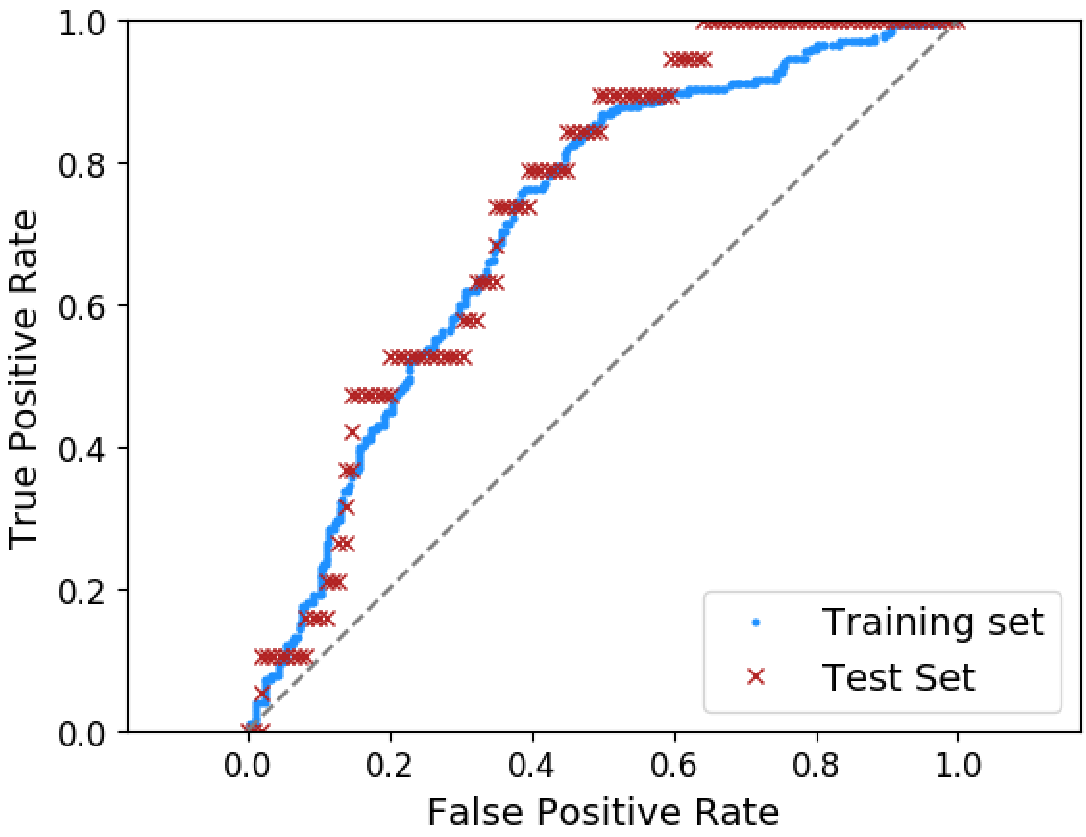

Supplement: Supplementary file 1 [file DataSheet1.PDF]
